# Measuring Adoption of Patient Priorities–Aligned Care Using Natural Language Processing of Electronic Health Records: Development and Validation of the Model

**DOI:** 10.2196/18756

**Published:** 2021-02-19

**Authors:** Javad Razjouyan, Jennifer Freytag, Lilian Dindo, Lea Kiefer, Edward Odom, Jaime Halaszynski, Jennifer W Silva, Aanand D Naik

**Affiliations:** 1 VA Health Services Research and Development Service Center for Innovations in Quality, Effectiveness and Safety Michael E DeBakey VA Medical Center Houston, TX United States; 2 Department of Medicine Baylor College of Medicine Houston, TX United States; 3 Big Data Scientist Training Enhancement Program (BD-STEP) VA Office of Research and Development Washington, DC United States; 4 Social Work Service Butler VA Health Care System Butler, PA United States; 5 VA National Social Work Program Office, Care Management and Social Work Patient Care Services Department of Veterans Affairs Washington, DC United States; 6 VA Tennessee Valley Healthcare System Nashville, TN United States; 7 VA Quality Scholars Coordinating Center IQuESt Michael E DeBakey VA Medical Center Houston, TX United States

**Keywords:** natural language processing, NLP, social work note, decision support, machine learning, pattern recognition, geriatric decision support system

## Abstract

**Background:**

Patient Priorities Care (PPC) is a model of care that aligns health care recommendations with priorities of older adults who have multiple chronic conditions. Following identification of patient priorities, this information is documented in the patient’s electronic health record (EHR).

**Objective:**

Our goal is to develop and validate a natural language processing (NLP) model that reliably documents when clinicians identify patient priorities (ie, values, outcome goals, and care preferences) within the EHR as a measure of PPC adoption.

**Methods:**

This is a retrospective analysis of unstructured National Veteran Health Administration EHR free-text notes using an NLP model. The data were sourced from 778 patient notes of 658 patients from encounters with 144 social workers in the primary care setting. Each patient’s free-text clinical note was reviewed by 2 independent reviewers for the presence of PPC language such as priorities, values, and goals. We developed an NLP model that utilized statistical machine learning approaches. The performance of the NLP model in training and validation with 10-fold cross-validation is reported via accuracy, recall, and precision in comparison to the chart review.

**Results:**

Of 778 notes, 589 (75.7%) were identified as containing PPC language (kappa=0.82, *P*<.001). The NLP model in the training stage had an accuracy of 0.98 (95% CI 0.98-0.99), a recall of 0.98 (95% CI 0.98-0.99), and precision of 0.98 (95% CI 0.97-1.00). The NLP model in the validation stage had an accuracy of 0.92 (95% CI 0.90-0.94), recall of 0.84 (95% CI 0.79-0.89), and precision of 0.84 (95% CI 0.77-0.91). In contrast, an approach using simple search terms for PPC only had a precision of 0.757.

**Conclusions:**

An automated NLP model can reliably measure with high precision, recall, and accuracy when clinicians document patient priorities as a key step in the adoption of PPC.

## Introduction

Older adults with multiple chronic conditions (MCC) frequently receive some of the most intensive and expensive health care, much of which is of uncertain benefit [1-3]. Care for these patients is often inconsistent and fragmented because the multiple specialists they see provide care based on single disease guidelines that do not take into account the complexities of MCC. Moreover, it often does not address what matters most to patients [1,2]. With input from patients, caregivers, clinicians, health system leaders, payers, and health care design experts, we developed Patient Priorities Care (PPC), which involves identifying, documenting, and providing care consistent with patients’ health priorities [4,5]. PPC is an intervention that aims to help clinicians provide health care to older patients with MCC that aligns with their priorities, which include their individual values (what matters most), desired outcome goals, and health care preferences [1-6].

In the PPC process, there are 3 key steps. First, a trained facilitator works with each patient to identify the patient’s health care priorities: their values, outcome goals, and health care preferences [1-3,5]. Values are the people, things, activities, and capabilities *that matter most* to a person, such as connecting with others, independence, enjoying life, and balancing quality and quantity of life [7,8]. Patient values provide the basis for setting specific, measurable, attainable, realistic, and timely outcome goals (SMART goals). Health care preferences are the activities a patient is willing (or not willing) to do to reach their outcome goals. The second key step in PPC involves documenting patient priorities in the patient’s electronic health record (EHR) [4,5]. This makes information about patient priorities available to different clinicians working with adults with MCC. In the final step of PPC, the clinician uses documented patient priorities to make treatment decisions that align with patient priorities [3].

Social workers frequently serve as facilitators in the PPC approach. Social workers use a holistic approach and encourage self-determination while promoting dignity and worth of the individual. They are also uniquely qualified to provide a wide array of quality social work interventions, including care coordination, case management, individual and group therapy, and supportive counseling for older adults with MCC. Social workers address social determinants of health and provide proactive interventions for social conditions that contribute to poor health outcomes. Social workers facilitate communication between the patient and health care provider and act as an advocate on behalf of the patient.

The PPC process thus relies on identifying and documenting patient priorities in the EHR so facilitators can share priorities with other clinicians. This documentation is done in the free text of individual visit notes. To measure adoption of PPC, the free text of clinician notes must be analyzed. Analyzing the free-text notes as yes (PPC present) or no (PPC not present) is entitled as text classification [9]. Although the gold standard for text classification is human chart review, it is tedious, time consuming, expensive, and potentially risks patient privacy [10]. To overcome these limitations, natural language processing (NLP) has been introduced as an alternative or adjunct to manual chart abstraction [10]. NLP refers to the computational methods of concepts, entities, events, and relations extraction from free text [11]. NLP algorithms are used in a wide spectrum of health care–related purposes such as identifying disease risk factors, evaluating efficiency of care and costs, and extracting information from clinical notes [12].

The first aim of this study was to develop an NLP algorithm capable of confirming social worker use of PPC in the free-text notes of patients’ EHRs. We hypothesized that the NLP model could confirm documentation of patient priorities in the free text of facilitator notes. We compared the performance of the NLP model with a chart‐reviewed reference standard by reporting performance measures (ie, accuracy, recall, specificity, precision, negative predictive value). To further evaluate the performance of the NLP algorithm, we compared its accuracy to a simple search terms approach. We also used the NLP algorithm to monitor the uptake of PPC among trained social workers following dedicated training sessions and track the persistence of PPC adoption over time.

## Methods

### Overview

To create the NLP model to assess the adoption of PPC, we used the notes of a cohort of social workers who were trained to be PPC clinicians over the course of 9 months. This cohort of social workers was hired through the VA Social Work in Patient Aligned Care Teams (PACT) Staffing Program. This program was developed in May 2016 and is the result of a partnership between the Veterans Health Administration (VHA) National Social Work Program Office and the Office of Rural Health. The goal of the program is to embed social workers in rural and highly rural areas to increase Veteran access to high-quality social work services. The innovative program supports the Office of Rural Health’s mission of breaking down barriers separating rural Veterans from high-quality care. Social workers are often one of the first clinicians a Veteran has contact with in the VHA. Social workers in this program provide interventions at numerous rural health VHA medical centers and outpatient clinics across the nation. Two cohorts of social workers were trained: 56 social workers from 5 VA health care systems in 2018 and 88 social workers from 12 VA health care systems in 2019. Social workers were trained to assess, evaluate, and select patients who were good candidates for PPC. Patients had to be older than 60 years of age and have at least two primary care encounters in a prior year. Social workers were trained to facilitate PPC conversations using patient and facilitator workbooks. Social workers were instructed to document all PPC-related interactions in the patient free-text note including all components of PPC. A PPC rubric was provided to social workers to document components of PPC: patient values, outcome goals, and health care preferences (see Multimedia Appendix 1). After reviewing the PPC template and related notes, we recognized that all notes must have 2 phrases: “goal” and “value.” Notes without these 2 expressions are not considered as PPC.

### Data Collection (Search Term Approach)

Notes of eligible patients were retrieved from the VHA Corporate Data Warehouse [13]. First, notes of all patients who visited with a PPC-trained social worker were retrieved from the Corporate Data Warehouse and included in a database. Notes were obtained from the first facilitated PPC visit through the end of the 2019 calendar year. The Baylor College of Medicine Institutional Review Board and the Veterans Affairs research service (IRB# H-41886) approved this study. Access to patients' data was granted through the Veterans Informatics and Computing Infrastructure.

### The Reference Standard

To develop the NLP algorithm, we extracted 778 notes from 658 patients who were seen by the social workers. We developed a reference standard for identifying PPC using a formal chart review. We provided complete copies of the notes from the 2018 and 2019 cohorts to 2 independent reviewers (EO and JF). The reviewers ensured the existence of language for values, outcome goals, and care preferences for each free-text note. Charts were labeled as 0 or 1; 0 refers to absence of documented patient priorities for the patient’s note, and 1 refers to documentation of priorities in free-text notes of patients. Any disagreement between reviewers was resolved by a third reviewer, a geriatrician involved in the development of PPC (AN). The agreement among reviewers is reported using the kappa statistic, and the significance level was set at *P<*.05; statistical analysis was conducted using SPSS software.

### NLP Algorithm

**Figure 1:**
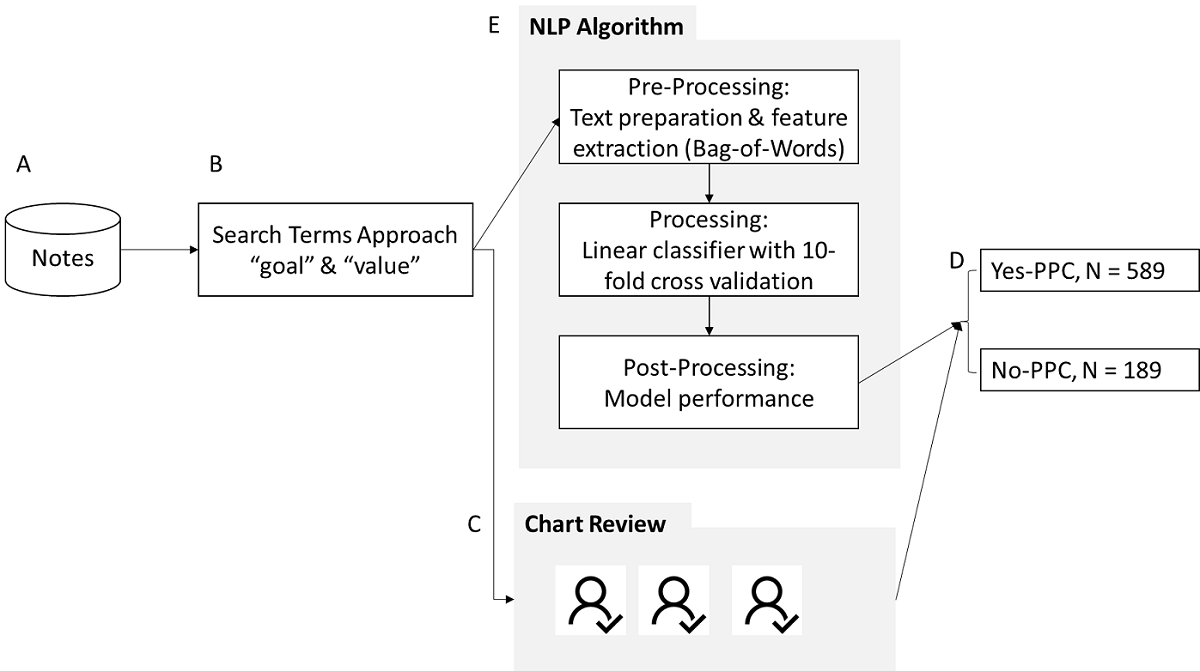
Process of developing the natural language processing (NLP) algorithm: (A) retrieving 106,505 notes from trained social workers after the first training workshop; (B) searching notes for existence of 2 expressions “goal” and “value,” which resulted into 778 notes; (C) review and resolution of disagreements; (D) labeling of notes as yes-Patient Priorities Care (PPC; 1) or no-PPC (0); (E) development of NLP algorithm.

The NLP algorithm was developed using 3 steps: preprocessing, processing, and postprocessing (Figure 1). In the preprocessing step, we cleaned the text, performed features extraction, and reduced the number of features to reduce the size of the feature space (dimensionality reduction). In the processing step, we developed a classification model to identify the yes-PPC from no-PPC notes. In postprocessing, we reported the performance models developed in the processing step and assessed the generalizability of the model.

#### Preprocessing, Cleaning the Text

We converted the stream of text into meaningful elements such as words, phrases, and symbols, called tokenization. We added the part of speech (eg, noun, verb, and pronoun) to each word. We removed stop words (eg, articles, conjunctions, and prepositions) and punctuation as they do not contain informational content. Words were normalized by reducing words to their word stem (stemming technique). For example, the words caregiver, caregivers, and caregiving would be reduced to “caregiv.“

#### Cleaning, Features Extraction, and Feature Reduction

To extract features from the text, we used “bag-of-words” (BoW). BoW (also known as a term-frequency counter) records the number of times that each individual word (unigram) appears in a document. We excluded unigrams with a frequency ≤2. These words must appear in the entire sentence related to goals and values <2 times (Multimedia Appendix 2 visualizes the BoW).

#### Processing Step, Developing a Classification Model

We fit a linear regression model to the BoW as an independent variable and the document label of 0 versus 1 as a dependent variable. To develop the model, we used K-fold cross-validation, with K=10. The goal of cross-validation is to test an approach's ability to predict new notes not used in training to avoid problems such as overfitting or selection bias [14]. In the 10-fold cross-validation, we randomly split our dataset into 10 equal sections [15]. We trained a model with 9 splits, and we validated the performance of the model on the remaining split. The process was repeated 10 times, and the average performance is reported.

#### Postprocessing Step

We tested each model using a validation cohort, using the model with the highest performance as compared to the chart review (validation of the model). To evaluate the performance of the NLP model in training, validation, and test phases, we used sensitivity or recall, specificity, accuracy, positive predictive value or precision, negative predictive value, and area under the curve [16,17]. To further validate the NLP approach, we compared its performance to an approach that used a simple search term assessment for the 2 expressions: “goal” and “value.”

### PPC Adoption

As a second objective, we evaluated the adoption of PPC among trained social workers. We counted the number of yes-PPC notes in each month, and we created a time series of notes identified by the PPC algorithm. The x axis shows the month, and the y axis shows the number of documented notes with PPC language in the EHR. This evaluation helped to understand the implementation of the PPC approach among social workers who received formal training and the persistence of PPC adoption over time.

## Results

### Data

We collected 778 notes that had “goal” and “value” within their text. These notes were collected from 658 patients from 17 VA facilities by 82 social workers. Mean patient age was 70.4 years (SD 15.2 years), 91.6% (603/658) were male, 23.4% (154/658) had all-cause-of-mortality recorded, 91.8% (604/658) were of “Not Hispanic or Latino” ethnicity, 71.9% (473/658) were White, 6.7% (44/658) were “Black or African American,” and 47.4% (312/658) were married.

The 2 reviewers disagreed on 54 notes and agreed on 724 (724/778, 93.1%) notes. The level of agreement after removing the chance effect was reduced to 82% (kappa=0.820, standard error 0.023, *P*<.001).

### Model Performance

The performance of the NLP model is shown in Table 1. The NLP model performance was calculated over notes (N=778) that had “goal” and “value” in their text. In the 10-fold cross-validation, the model validation reached an average of 0.92 (95% CI 0.90-0.94) for accuracy, 0.84 (95% CI 0.79-0.89) for sensitivity, 0.95 (95% CI 0.92-0.97) for specificity, 0.84 (95% CI 0.77-0.91) for the positive predictive value, and 0.95 (95% CI 0.93-0.96) for the negative predictive value.

**Table 1:** Comparison of chart-reviewed, free-text notes and natural language processing (NLP) model for the training and validation.

Parameters	Training, mean (95% CI)^a^	Validation, mean (95% CI)^a^
Accuracy	0.98 (0.98-0.99)	0.92 (0.90-0.94)
Sensitivity or recall	0.98 (0.98-0.99)	0.84 (0.79-0.89)
Specificity	0.99 (0.98-0.99)	0.95 (0.92-0.97)
Positive predictive values or precision	0.98 (0.97-1.00)	0.84 (0.77-0.91)
Negative predictive value	0.99 (0.98-0.99)	0.95 (0.93-0.96)

^a^Reported for 10-fold cross validation.

In contrast, an approach using simple search terms (“goal” and “value”) applied to large text performed less well. The total number of notes that the social workers produced was 106,505. Table 2 describes the precision of this model. The precision of the NLP model was >0.99, while the precision of the simple search terms was 0.7571. In the other words, the simple search terms method falsely recognized 1 out of 4 notes as PPC.

**Table 2:** Comparison of the natural language processing (NLP) model and simple search terms on all 106,505 notes.

Parameters	Search terms	NLP model
Accuracy	0.9982	0.9999
Sensitivity or recall	1.0000	0.9999
Specificity	0.9982	0.9983
Positive predictive values or precision	0.7571	1.0000
Negative predictive value	1.0000	0.9849

**Figure 2:**
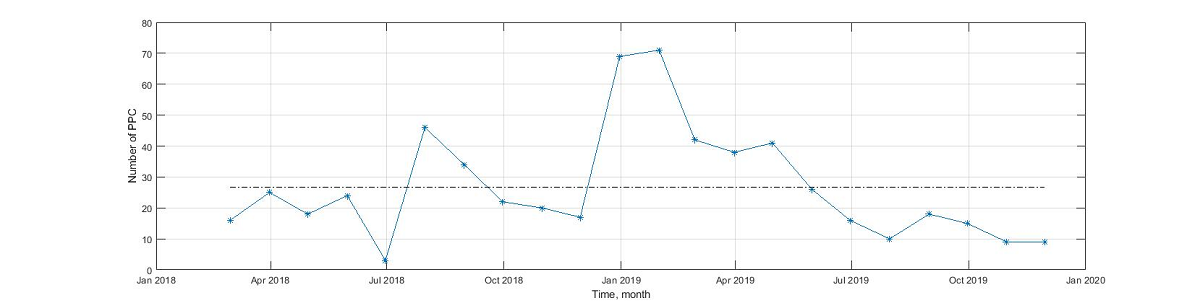
Monthly report of the number of Patient Priorities Care (PPC) encounters documented in the patients’ free-text notes in the Veterans Administration (VA) electronic health records; the 2 peaks are associated with training of social workers to use PPC.

Figure 2 describes the adoption of PPC within the notes of trained social workers over time. The 2 peaks represent the times immediately following training of social workers to use PPC. Figure 2 also demonstrates that several months following the cessation of training, the persistence of PPC adoption declined. The NLP algorithm was useful in tracking PPC adoption over time.

## Discussion

### Principal Findings

Our results demonstrate that an NLP algorithm was highly accurate in identifying patients’ free-text notes with PPC language. Such a design has shown promise in previous work identifying risk factors for heart disease [18]. The NLP model had a high performance of 0.84 in validation, and its precision was higher than an approach using only simple search terms. The NLP algorithm was also able to capture peaks in PPC adoption following social worker training and the eventual decline in PPC adoption over time. Such a tool may be used to monitor the overall implementation of interventions like PPC among trained social workers and to adjust the training process.

While this NLP model performed well, model performance (precision and recall) could improve with the use of more complex strategies such as utilizing bigram or n-gram BoW, other sophisticated feature extractions (eg, graph mining [19]), and nonlinear models (eg, deep learning [20]). To reduce the computational cost of developing and implementing a model to test the use of PPC by trained facilitators, we propose the NLP model as a method that reduces the time and cost with other methods of assessment. Further development of this model will offer assessment with more precision. Despite these limitations, the NLP model performed better than the simple search term method, which had a 1-in-4 notes rate of misclassification.

This study opens 3 new avenues for further investigation. By identifying PPC notes, we can longitudinally assess care for patients throughout VA facilities and understand changes in health care delivered after PPC encounters [6,21]. We can identify patterns and consistency in how social workers and other clinicians document patient priorities over the course of multiple interactions with patients [3]. By further expanding the NLP algorithm, we can also examine common priorities among our patient population and increase clinician, patient, and caregiver awareness of priorities recorded in the EHR. For successful implementation of PPC, we must assess other factors that play roles in the successful implementation of PPC, such as resource availability, adoption of PPC on a provider and facility level, and readiness of clinicians for implementation [4]. We can also use sociodemographic data to pinpoint the ways individual characteristics of patients might influence the implementation of PPC by clinicians. We can also provide individualized feedback to each clinician to enhance their patient engagement process. Using this algorithm, we can measure long-term outcomes of interest, such as mortality and independent living.

### Implications

This study achieved its overall objective of developing a simple NLP model to identify free-text notes that show evidence of documented patient priorities as part of the PPC process. Our NLP model complements PPC by measuring the extent to which social workers trained in PPC identify and document patient priorities. The NLP model can enable trainers to provide feedback on the implementation of PPC in the EHR. Additionally, this method can analyze the free text of any note for documentation of patient priorities generally in an effort to link those documented priorities with many patient outcomes without requiring labor-intensive methods for individual chart review.

## Appendix

Multimedia Appendix 1 Note template provided to the social workers during online training.

Multimedia Appendix 2 Visualization of each word in the Patient Priorities Care (PPC) notes, with the importance based on frequency.

## Abbreviations

BoW: bag-of-words
EHR: electronic health record
MCC: multiple chronic conditions
NLP: natural language processing
PACT: Patient Aligned Care Teams
PPC: Patient Priorities Care
VHA: Veterans Health Administration
