# Supplementary figures and images for: Measuring Adoption of Patient Priorities–Aligned Care Using Natural Language Processing of Electronic Health Records: Development and Validation of the Model
Source: JMIR Med Inform. 2021 Feb 19;9(2):e18756. doi: 10.2196/18756 (PMC7935648; doi:10.2196/18756)

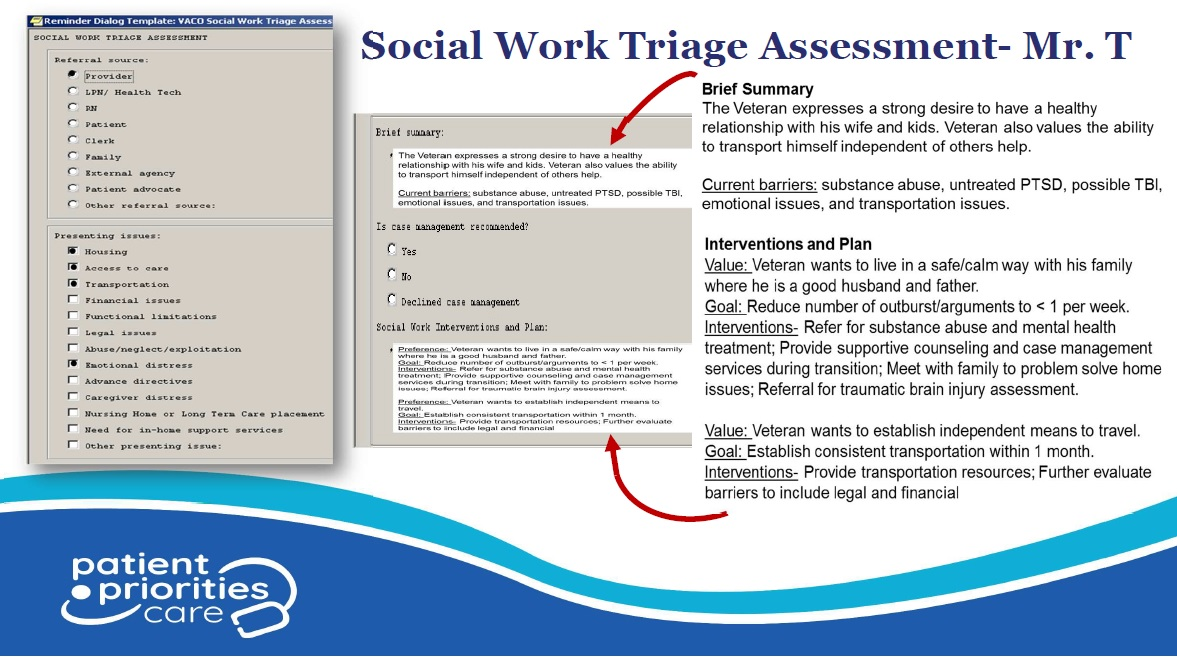

Supplement: Multimedia Appendix 1 [file medinform_v9i2e18756_app1.png]

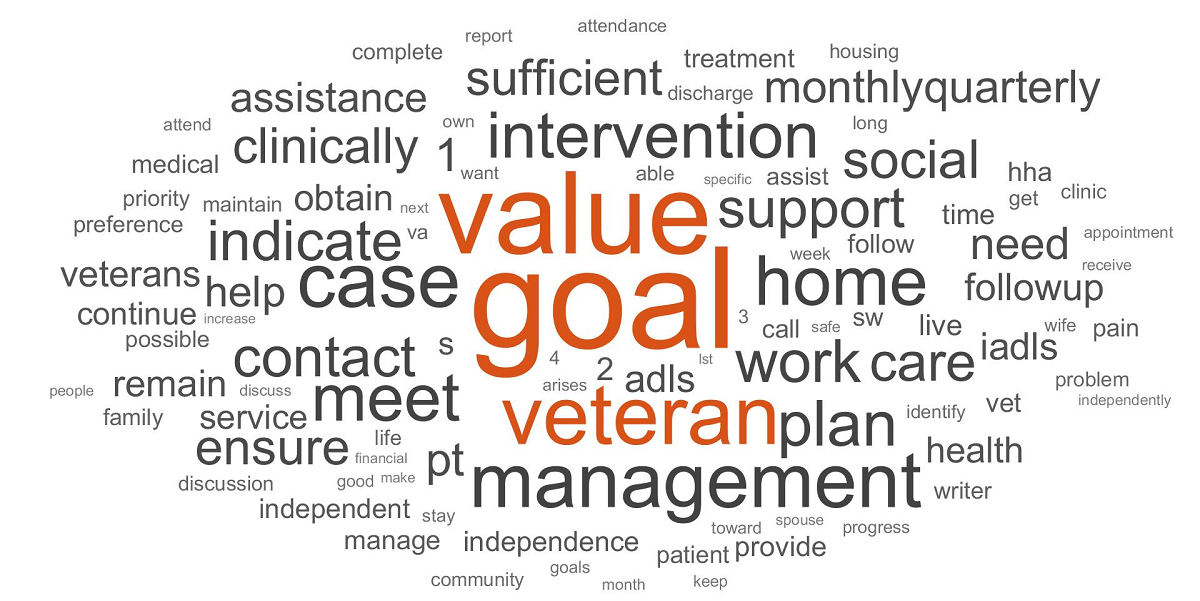

Supplement: Multimedia Appendix 2 [file medinform_v9i2e18756_app2.png]
